# It turned into something else: patients’ long-term experiences of transitions to or from telepsychotherapy during the COVID-19 pandemic

**DOI:** 10.3389/fpsyg.2023.1142233

**Published:** 2023-05-12

**Authors:** Camilla von Below, Jenny Bergsten, Therése Midbris, Björn Philips, Andrzej Werbart

**Affiliations:** Stockholm University, Stockholm, Sweden

**Keywords:** remote psychotherapy, online therapy, communication technology, patient experiences, therapeutic boundaries, therapeutic relationship, thematic analysis

## Abstract

**Introduction:**

The shift from in-person therapy to telepsychotherapy during the COVID-19 pandemic was unprepared for, sudden, and inevitable. This study explored patients’ long-term experiences of transitions to telepsychotherapy and back to the office.

**Methods:**

Data were collected approximately two years after the declaration of COVID-19 as a pandemic. Eleven patients were interviewed (nine women and two men, aged 28 to 56, six in psychodynamic psychotherapy, five in CBT). Treatments switched between in-person and video/telephone sessions. Interview transcripts were analyzed applying the qualitative methodology of inductive thematic analysis.

**Results:**

(1) The patients experienced the process in telepsychotherapy as impeded. Interventions were difficult to understand and lost impact. Routines surrounding the therapy sessions were lost. Conversations were less serious and lost direction. (2) Understanding was made more difficult when the nuances of non-verbal communication were lost. (3) The emotional relationship was altered. Remote therapy was perceived as something different from regular therapy, and once back in the therapy room, the patients felt that therapy started anew. The emotional presence was experienced as weakened, but some of the patients found expressing their feelings easier in the absence of bodily co-presence. According to the patients, in-person presence contributed to their security and trust, whereas they felt that the therapists were different when working remotely, behaving in a more easygoing and familiar way, as well as more solution-focused, supportive and unprofessional, less understanding and less therapeutic. Despite this, (4) telepsychotherapy also gave the patients an opportunity to take therapy with them into their everyday lives.

**Discussion:**

The results suggest that in the long run, remote psychotherapy was seen as a good enough alternative when needed. The present study indicates that format alternations have an impact on which interventions can be implemented, which can have important implications for psychotherapy training and supervision in an era when telepsychotherapy is becoming increasingly common.

## Introduction

Telepsychotherapy enabled patients to continue to access psychotherapy during the COVID-19 pandemic. However, neither patients nor therapists were prepared for or expected such a forced change of format from sessions in the room to video or telephone sessions, and research into the effects of the shift has accumulated in the wake of the pandemic. Nevertheless, different forms of remote psychotherapeutic treatments have been used for a long time and have been increasingly considered to be an acceptable alternative to conventional settings, often working equally well for different types of psychological problems and of treatments (see below). These can be designed as guided self-help with minimal and asynchronous communication with the therapist (Cuijpers et al., 2010), or as video-mediated treatment, based on synchronous online communication, sometimes called ‘videoconferencing’ (Simpson et al., 2005; Connolly et al., 2020; Fisher et al., 2021). Nowadays, remote psychotherapeutic treatments are included under such umbrella terms as ‘telemedicine’ or ‘telemental health’ (Hilty et al., 2013; Connolly et al., 2020), ‘telepsychology’ (American Psychological Association, 2013) or ‘telepsychotherapy’ (McMullin et al., 2020; Poletti et al., 2021), and there is a lack of consensus on terminology (Smoktunowicz et al., 2020). ‘Hybrid therapy’ is a treatment in which the setting alters between in-person and teletherapy. In the present study, we use the terms ‘remote therapy’ or ‘telepsychotherapy’; however, when referring to other studies, we follow the terms used by the respective authors.

The use of communication technology has been discussed in the psychoanalytic tradition since the aftermath of World War II, when Saul (1951) drew attention to the use of the telephone as a technical aid helpful in psychoanalysis with some patients. Following rapid technological developments, this discourse expanded significantly. Carlino (2011) argued for the evolution of psychoanalytic theory and practice in the digital era, when teleanalysis may be the treatment of choice for many people. However, relationships and communication in cyberspace are seen as fundamentally different from those happening in a shared physical space (Sabbadini, 2014). A specific concern among psychoanalysts is the fate of the body in the virtual space (Carlino, 2011; Lemma, 2015). The cross-modal interaction between the senses gets lost without physical proximity (Bayles, 2012). The lack of the concrete presence of people’s bodies in a room makes it necessary to create an illusion of presence, i.e., to establish ‘telepresence’, which is possible when communication technology works (Essig and Russell, 2021). Furthermore, there is a need to adapt interventions to remote treatments (Scharff, 2012; Fisher et al., 2021).

Comparing the remote and in-person settings, it is important to notice the difference between deliberate teleclinical practice and rapid, unprepared transitions to telepsychotherapy due to restrictions following the outbreak of the COVID-19 pandemic. A meta-analysis of the efficacy of in-person and video-delivered psychotherapy (Fernandez et al., 2021) showed negligible differences between the two formats. However, improvements in video-delivered psychotherapy were most manifest in cognitive behavioral therapy (CBT) addressing anxiety, depression, or post-traumatic stress disorder (PTSD). In a study applying the ‘Multitheoretical List of Therapeutic Interventions’ (MULTI-30; Probst et al., 2021), therapists rated all interventions as more typical for in-person therapy than remote therapy, whereas patients regarded psychodynamic, process-experiential, and cognitive interventions as more typical for in-person therapy, indicating that therapeutic interventions differ between in-person and remote therapy. Poletti et al. (2021) concluded in a review of 18 studies that telepsychotherapy is as effective for depression, anxiety and PTSD as in-person therapy of different orientations, although therapists and patients might experience initial skepticism and technical difficulties. Previously, a systematic literature review (Backhaus et al., 2012) had found that video-mediated remote therapy had similar clinical outcomes as in-person therapy for anxiety and depression, PTSD, obsessive–compulsive disorder, panic disorder, and social phobia. However, psychotherapy for patients with pain seemed to be more efficient in person (Chavooshi et al., 2017). A systematic review of 24 studies (Margherita et al., 2022) showed that online group interventions during the COVID-19 pandemic were effective in reducing psychological distress and increasing psychological interpersonal resources. An online survey of 281 Italian licensed psychotherapists in the early phase of the pandemic (Mancinelli et al., 2021) showed that the therapists forced to shift to online work were able to preserve their positive professional self-perception. However, they reported being much more conversational and directive in remote sessions, possibly trying to compensate for the physical distance. Furthermore, they felt more fatigued not having access to non-verbal cues in remote sessions. In another online survey among 507 Italian psychotherapists with different orientations (Cantone et al., 2021), the participants reported critical issues with remote work, such as the need for greater flexibility, greater attention, and greater concentration, resulting in greater fatigue. Furthermore, most of them discovered that remote work, while more suitable for some patients, may be inappropriate for others. The authors concluded that the psychotherapists seemed to have difficulty adjusting their technical repertoire to the shift to a remote setting. A qualitative study of 15 psychologists’ experiences of telepsychotherapy within the Irish Mental Health System (Reilly et al., 2022) revealed that the participants experienced loss of control over therapeutic boundaries and of non-verbal cues, had to work much harder to establish a bond with their clients, and lacked professional support in the transition. In a critical commentary, Smith et al. (2022) concluded that despite studies demonstrating the effectiveness of video-mediated therapy, the current evidence base is still limited, and that this therapy setting might not suit all patients and all therapeutic orientations. Further research might conclude that telepsychotherapy can be more suitable for patients with certain non-diagnostic characteristics and personality factors. Accordingly, recent studies (Aafjes-Van Doorn et al., 2021a,b; Békés and Aafjes-van Doorn, 2022) found that patients with attachment anxiety experienced more distress in remote therapy during the COVID-19 pandemic. The researchers concluded that the working alliance and therapeutic agency may differ in importance for patients depending on their attachment style, since the therapeutic relationship and emotional closeness is of greater importance for patients with anxious attachment.

The research focusing on the psychotherapists’ experiences of the rapid and unprepared shift from in-person therapy to telepsychotherapy during the COVID-19 pandemic, shows that the the new format proved challenging. The pandemic in itself can be viewed as a shared traumatic experience that put patients and therapists in the same uncertain and health-threatening position as the virus itself (Nuttman-Shwartz and Shaul, 2021), thus changing the therapist role. Important features of therapy, such as non-verbal communication and body language, as well as the finely-tuned adjustments that therapists make in turn-taking and sensitivity to create a therapeutic alliance, were lost with video and phone or had to be modified. The assessment of patient difficulties was also more difficult to do remotely (Feijt et al., 2020; Békés et al., 2021; Fisher et al., 2021; Lin et al., 2021; James et al., 2022; Khan et al., 2022). The technological solutions were unfamiliar to many therapists and thus made them feel uncertain to begin with (Békés et al., 2021). The therapy room’s safe space and confidentiality did not come across as easily on screen or telephone when patients had to find their own room for their remote therapy sessions (Ahlström et al., 2022). In therapists’ experience, this had a negative effect on the therapeutic alliance and on patient adherence to the treatment (Lin et al., 2021). The sudden change of format has led therapists to modify their interventions.

### The current study

Research into how patients experience the change of format and the adjustment to interventions is not yet fully developed (Farber and Ort, 2022). Even less research has been done on the experience of changing to telepsychotherapy and then back again to in-person-therapy, which will probably be more common after the pandemic, when patients and therapists are used to the remote format and need it from time to time. However, there are some studies indicating that both patients and therapists may experience an advantage with hybrid settings, i.e., that sessions within the same therapy can be either remote or in-person (Sperandeo et al., 2021; Leuchtenberg et al., 2022).

Patients might differ in their ability to adapt over time to telepsychotherapy and to benefit from the altered format, as well as in how they experience transitions to and from telepsychotherapy. In a previous study (Werbart et al., 2022), we explored patient experiences of the transition to telepsychotherapy shortly after the onset of the COVID-19 pandemic. The present study was aimed at investigating patients’ long-term experiences of transitions to telepsychotherapy and eventually back to the office. The research questions were: What factors are perceived by patients as contributing to their both positive and negative long-term experiences of transitions to remote therapy? What are the positive and negative aspects experienced by patients in relation to a possible return to in-person setting?

## Materials and methods

### Procedure and participants

Inclusion criteria for the present study were: experience in undergoing psychotherapy with a licensed psychologist or licensed psychotherapist with a frequency of at least once a week and a duration of no less than 4 weeks before transition to or from telepsychotherapy due to the COVID-19 pandemic. Eleven participants were included. One of them had previously taken part in a study focusing on short-term experiences of the transition from in-person sessions to telepsychotherapy (Werbart et al., 2022), which the present study was intended to follow up. Additional participants were recruited *via* social media, and 28 people registered an interest in participating. Contact was made with all of them, and 10 could be included. Of the remaining 18, the majority did not respond to further contact and in two cases their therapies did not meet the inclusion criteria.

All the 11 participants met the inclusion criteria and gave their informed consent to participate in the study. The platform Survey and Report, provided by Stockholm University, was used to collect the consent. The age of the participants ranged from 28 to 56 years (*M* = 39.8). Nine of the participants were women and two were men. No questions were asked about the participants’ presenting complaints, but the interview responses indicated different levels of severity of psychological difficulties (such as depression and anxiety). Time in therapy prior to the first transition varied between two and 120 months, and the therapy duration was between 2.5 and 27 months. Five participants were in cognitive-behavioral therapy, a further five in psychodynamic therapy, and one participant was not sure of the therapeutic orientation. All but one of the participants had started their therapy in a conventional in-person setting, except one who had begun therapy remotely on video. Three of the participants had their remote sessions over the telephone, seven of them had their remote session mostly on video but with occasional sessions on the phone, whereas one participant had had a period of 4 months on the phone before switching to video and then transitioning back to in-person sessions. Eight of the participants had experience of two transitions, from the conventional in-person setting to telepsychotherapy and back again to the in-person setting; three of the participants had experience of one transition; in two cases to telepsychotherapy and in one case from remote sessions to an in-person setting (See Table 1).

**Table 1:** Participant characteristics: therapy type (PDT = psychodynamic psychotherapy and CBT = cognitive-behavioral therapy), time in therapy before the first transition, length of therapy, communication technology, and number of transitions.

Participant #	Therapy type	Time before the first transition	Length of therapy	Communication technology	Number of transitions
1	PDT	5 months	18 months	Video	2
2	PDT	5 months	12 months	Video	2
3	PDT	2 months	4 months	Telephone	1
4	CBT	2 months	27 months	Telephone/video	3
5	PDT	5 months	26 months	Telephone	2
6	PDT	15 months	24 months	Video	2
7	CBT	2 months	2.5 months	Video	1
8	CBT	18 months	NS	Video	2
9	NS	120 months	Ongoing	Telephone	1
10	CBT	6 months	18 months	Telephone	2
11*	CBT	5 months	7 months	Video	2

NS, Not specified.

* Therapy started remotely. Time in therapy prior to the first transition to remote setting.

### Data collection

Data were collected in spring 2022 through semi-structured interviews conducted online. The interviews lasted for about 45 min and were audio-recorded using the Zoom platform’s audio. The interviewers were the second and the third author, who at the time of the study were students in the final semester of the Swedish three-year advanced psychotherapy training program leading to a Swedish psychotherapist license. Both interviewers conducted five or six interviews, and they had previous clinical experience working with psychotherapy patients switching from conventional in-person settings to remote sessions.

The interview protocol was aimed at collecting narratives around long-term, both positive and negative, experiences of transitions to telepsychotherapy or in the opposite direction. The questions were open and encouraged participants to express themselves freely. The participants were asked how the transitions had affected the patient-therapist relationship, the therapy process, and the experienced outcome of therapy. The interview questions covered the more hindering and more helpful aspects of the transitions and how the experiences had changed over time. Participants were encouraged to elaborate on their answers and give concrete examples. Key questions included: How did you and your therapist decide to switch to remote therapy or to therapy at the therapist’s clinic? How did you experience this transition (positive and negative experiences, concrete examples)? How did the transition affect the therapy? How open did you feel in the therapy? How well were you able to profit from the therapy? What were your feelings about the therapy? How was your relationship with the therapist? What is your view of the therapist? What were your feelings concerning trust toward the therapist? How did these experiences affect you? How did your experience of remote therapy and therapy at the therapist’s clinic change over time?

### Analysis

The interview data were analyzed by the second and third author, with supervision from the first author, following Braun and Clarke’s (2006, 2013, 2022) six steps of inductive thematic analysis. Step one included familiarizing themselves with the data when transcribing the interviews, reading through the material, and noting down their own thoughts and ideas. Step two was initial coding of the interview transcripts. To ensure an inductive stance, the initial coding was made by the person who had not conducted the interview, i.e., author two or three. In step three, the second and third authors worked jointly to group codes into preliminary themes and discussed these. Together, they gathered the preliminary themes into three main themes. In step four, the main themes were examined in relation to associated codes and relevant sections of the transcripts, and the relationship between themes was explored. Sub-themes were merged and delineated. Moving back and forth between the whole data set, the coded extracts, and the emerging thematic structure, represented in mind-maps, resulted in the fourth main theme. In step five the themes were defined and described, capturing the essence and what was specific for each theme. The themes were given final headings, and a last review was done to ensure that the thematic structure represented the overall experiences of the participants. Steps three to five were audited by and conducted in collaboration with the first and fifth author.

Frequency of participants contributing to each theme was examined and reported following the guidelines of Hill et al. (2005). Themes represented by 10–11 participants were labeled *general*, those by 6–9 participants were labeled *typical, and those by* 2–5 participants were labeled *variant.*

## Results

Thematic analysis resulted in four main themes and six sub-themes (Table 2). All themes are presented below and illustrated by verbatim quotations from the interviews. The main themes (1) *Impeded Process* and (2) *Altered Emotional Relationship* were categorized as general, whereas the main themes (3) *Restricted Understanding* and (4) *New Opportunities* were categorized as typical. Implicit in all the themes is the predominant, shared experience that the content of the therapeutic encounters and the therapeutic work were perceived as different, less efficient, even if telepsychotherapy also opened new prospects. Accordingly, the core, overarching theme is formulated as *It Turned into Something Else*, also explicitly expressed in the following quotes:

… but then it’s different to sit alone at home and think out loud than to meet someone in a room.

… experience that we are people who talk on the phone … and that is something else for me [laughs] than going to therapy, like that.

**Table 2:** Themes and sub-themes in the participants’ long-term experiences of transitions between telepsychotherapy and the in-person setting.

Theme	Frequency (*n* = 11)	Label
1. Impeded process	10	General
°1.1. Availability at the expense of efficiency	10	General
°1.2. Lost accuracy and impact of interventions	7	Typical
°1.3. Lost routines and rituals	7	Typical
2. Restricted understanding	8	Typical
3. Altered emotional relationship	11	General
°3.1. Distance for better or worse	11	General
°3.2. In-person presence contributes to security and trust	9	Typical
°3.3. The therapist seems different at a distance	8	Typical
4. New opportunities	8	Typical

Frequencies of participants in each theme and sub-theme (labeled following Hill et al., 2005): General = 10–11; Typical = 6–9; Variant = 2–5.

### 1. Impeded process

This general theme captures the participants’ experience of an inhibited or stalled process during remote sessions. The purpose, aim and quality of therapy were affected and hence negatively influenced the experienced efficiency.

#### 1.1. Availability at the expense of efficiency

Generally, the participants mentioned the availability of remote therapy as a positive aspect. Even if they lived at a distance or could not travel, the continuity could still be intact. According to one participant: “Otherwise it would not be possible. Without that technology we would have had to cancel and that would have made me feel worse.” Another participant caught two sides of the same coin:

Mostly remote therapy was negative but at the same time I’m really grateful for this opportunity. Otherwise, I would not have had any therapy at all… and this is better, it is better than nothing.

However, the participants experienced that this availability came with a cost. They experienced therapy as less serious and more superficial: “Somehow, for me, it felt like everything we said became less rooted in me.” The purpose and aim of the treatment were perceived as vague and less effective, more like an ordinary conversation than psychotherapy: “It was like the therapeutic process became more difficult and it became more difficult to reach and to get into such a state and not just a conversation about what has happened…” Something seemed to be lacking: “This [back to the therapist’s office] feels like therapy, and what was before was a kind of trudging along at best, more like support.” The participants also found it more difficult to maintain focus and to concentrate in remote sessions: “It took a lot of concentration and I felt fatigued by it.” Fatigue and passivity made therapy less intense: “It wasn’t as effective. It was hard to understand what we were doing. It wasn’t clear for me what the purpose of our sessions was, not as clear as it is now when we are back in the room.” Once back to the therapy room, the participants felt that therapy started from the beginning again: “It felt like I had to find a way to relate to him the first time I got there IRL.”

#### 1.2. Lost accuracy and impact of interventions

Typically, the participants reported that some therapeutic interventions were no longer possible or became more difficult to receive in remote sessions. They felt that some essential parts of the therapeutic work could not be done remotely as it would mean too much anxiety without sufficient support: “I feel I cannot let it out, as I am afraid to lose it, so to speak.” The assistance of a therapist present in the same room was experienced as necessary for working with trauma, dissociation, and close relationships: “In therapy, it’s in the relationship with the therapist that the work is done and … yes … I think that was more difficult…” Even when the participant tried to engage with the therapist as much as in therapy in the room, it became difficult:

When it is remote, it feels a bit unreal and does not matter if it is this or that, if I cry, whatever… but in the room it feels more important because it is a real person… so I want to share and I want to be myself, but it is more difficult on screen.

Furthermore, the use of therapeutic aids, such as a whiteboard or handouts, was more difficult when presented on screen. Homework was not as easy: “Homework got harder, harder to let it take time, you did not work through the feelings as much when they came up, without direction, no clear themes and no depth.” Therapeutic accuracy was lacking in other ways too:

I felt like he would catch me [in the room] and not let me get away with being vague and just talk on, but he would say that I was avoiding the work, but now [in remote sessions] I felt he chatted on, and we both avoided the work. I would have needed that he caught me and asked kind of, ‘why are we here, what do you want to do?’

#### 1.3. Lost routines and rituals

When routines, such as traveling to and from the clinic or sitting in the waiting room, disappeared in remote sessions, the participants typically experienced less time for reflection and processing. They also felt that the working through and thinking that took place in the remote sessions did not have the same quality: “Well, I guess that it felt more difficult to stay in touch with things that had been said. To let it take place in my everyday life …” The start and ending of the sessions became diffuse: “It was like a small ceremony, I guess you could say.… There’s also, maybe, a bigger difference before and after.” In retrospect, with the experience of both remote sessions and in-person-therapy, this difference became even clearer for the participants:

Then I think you lose your own ability to reflect, because it becomes much easier to… you schedule your work meetings and then you have an hour of therapy and then 3 min after the therapy ends, you have switched to something else…

### 2. Restricted understanding

Typically, the participants expressed that such vital aspects of non-verbal communication as body language, eye contact and tone of voice were lost in remote therapy. They found it more difficult to communicate to their therapists how they felt. The participants typically experienced that remote therapy negatively affected the ability to read between the lines: “I would say that there was a lot of misunderstanding, that I did not understand what she meant and that she did not understand what I meant.” Thus, remote sessions increased the risk of misunderstanding and misinterpretation. Back in the room it became a challenge to re-relate to body language: “Suddenly there is a lot more than a face on screen; there is body language and kind of… other information that you perceive.” Another participant also remarked on the difference in communication in remote sessions and therapy in the clinic: “There is so much communication through your body and voice that disappears in a video session and when you meet again, so to speak, it becomes much more tangible, and it shows itself.” Facial expressions were visible on screen but could be difficult to interpret in the absence of other non-verbal cues and co-presence: “She did not experience what I felt when we were not in the same room… She had no way of understanding how I was reacting and not reacting only seeing my face.” On the other hand, lack of eye contact could be experienced as beneficial by participants who found it easier to address matters verbally. One participant said that once back in the room she acknowledged how important eye contact was and how it had been missing in remote therapy: “If I say something [in the room] and I do not know what I feel, I can see how she reacts, I can read in her eyes and see my feelings in hers.” According to another participant, remote sessions work better if you were already familiar with your body language and how the therapist works. This participant stressed that remote therapy demands a higher degree of self-awareness to make oneself understood, and that self-awareness is easier attained in the bodily co-presence with the therapist.

### 3. Altered emotional relationship

The emotional closeness to the therapist was generally described as altered in remote sessions. The participants felt that the therapist was changed by the remote contact in a way that affected the safety and trust that had been created. This in turn seemed to affect the emotional content in the therapy.

#### 3.1 Distance for better or worse

This general theme captures the experience of the importance of maintaining contact and the relationship with the therapist during remote sessions. The emotional closeness and content of therapy were affected. The regulation of distance in remote therapy contributed to a difference in the therapeutic work, which some patients saw as positive and others as negative. Whereas one participant reported that remote therapy made it easier to avoid painful feelings, another reported that the stress increased and self-regulation became necessary to prevent a dissociative state. The quality of the relationship with the therapist seemed more important for the participants than the in-person or remote format of the therapy.

A general experience was that emotional closeness diminished during remote therapy. Some participants saw this as positive, since they sometimes preferred more distance. It could be easier to carry out emotionally demanding tasks and to be open when experiencing closeness in remote contact. The participants could feel less shy and thus find it easier to communicate difficulties, express emotions and avoid feelings of shame. For some, it also contributed to experiencing fewer feelings, which they considered positive. For one participant, the distance gave a feeling of independence and self-confidence, as the therapist seemed to trust the participant’s own ability. This participant also experienced it as positive that the remote therapy did not lead to a dependency and helped them to let go when approaching the termination of the sessions. The more distanced remote contact with the therapist was interpreted as something positive by this participant.

It was quite nice being able to just sit behind my screen, and I could choose whether she got to see my face or how close she could approach me. And in some way, I think it was rather nice that you could choose in a way, at the same time as I knew that it would have been a better challenge for me to actually see each other because that challenges me more.

Other participants found it difficult to stay in tune with emotions, acknowledge their feelings and dare to express them in remote sessions. One participant reported not daring to be angry in remote therapy, feeling sad instead. The participants experienced feelings of not being taken seriously or not being validated enough in remote sessions. The relational contact in the remote setting could be experienced as impersonal, anonymous, and less intimate, awakening yearnings to go back to the therapy room. The distance could also lead to a lack of feeling co-presence with the therapist and to a struggle to maintain one’s feeling of presence during the sessions.

#### 3.2. In-person presence contributes to security and trust

Typically, the participants considered it important to start the therapeutic process in person. It contributed to feelings of safety and trust, facilitating the transition to remote therapy. One of the participants started therapy remotely and described difficulties in trust and safety before meeting in person. Some participants did not experience any difference in trust and safety in remote therapy, since the trust created in the in-person sessions was carried with them into remote therapy. The participants experienced that the therapist from the therapy room remained real within them, which made the transition to remote sessions safe: “The trust that we have built up, it is still there, it is not the one that is destroyed.” For other patients, the safety that had been grounded in the co-presence with the therapist in the room decreased in remote therapy. This affected their ability to be emotional and open: “This energy, who am I talking to, where is he sitting, so what … how is that … does it feel safe?”

#### 3.3. The therapist seems different at a distance

Typically, the participants experienced that the therapist and the therapist role changed in remote sessions. The therapist became more light-hearted, easygoing and casual, the therapist’s private life became more visible, the therapist’s and patient’s roles were loosened, and the relationship was perceived as more friendly. Some experienced this as positive and that self-disclosure became easier when the therapist also was more open: “So, he talked about himself much more when it wasn’t exposure therapy. Yes, I think that all these things, they make me feel a little more comfortable and willing.” Others experienced this as a loss: “Then it was a bit like “Hello,” “Hello,” and “Hi,” “Hi;” there was a different tone a bit, in his voice and in my voice; we were on a different forum.. more private forum.” The therapists seemed more solution-focused, flexible and available in remote sessions, which could be seen as a sign of more caring. In remote contact, the participants could meet their therapist even when the therapist was sick. Some of the participants appreciated that the therapist offered this; others perceived the therapist as less professional when he or she conducted therapy even when ill. Also, learning private things about the therapist was a burden.

### 4. New opportunities

Typically, the participants felt that remote sessions created new opportunities. Therapy could continue despite isolation, illness or other duties:

People might have been isolated and did not do much and for me the situation was extreme, as I had been very ill for some time and did not see anyone except those who helped me clean… I had not left my flat for weeks. So for me it was very nice to have a task like this [remote sessions] to do.

The participants could bring the therapist with them in different situations, like on trips, after a move or in especially difficult situations when the need for therapy was increased:

At that moment my video session had just started, so I got online, and my therapist talked to me until the ambulance arrived, and she would not have been able to do this otherwise … you can meet the person where they are. If I had had a session at the clinic, that would have been cancelled.

Attending remote sessions from home also gave patients the opportunity to create new routines surrounding therapy, such as taking their own therapy notes on the computer. Being able to take therapy into their real lives could give a feeling of freedom: “The feeling of freedom and that maybe.. I mean, that you go far away but you still feel that you can have conversations.” The remote format could give access to a wider range of therapies, despite patients living a long way from the therapist’s office. Transition to remote sessions could shake up the therapeutic relationship, leading to challenges that could be experienced as new possibilities for personal growth. Remote sessions could be helpful in the process of ending therapy, giving an opportunity to get used to no longer meeting the therapist, and to become more independent.

## Discussion

To sum up our main findings: The patients experienced that the remote sessions provided availability at the expense of efficiency. The therapists’ interventions were more difficult to receive and lost some of their impact. Interventions including a whiteboard or textual material could not be done as usual, and the therapist’s distance hindered focus on trauma. The therapeutic process went more slowly, and the treatments were experienced as less efficient. Several routines and rituals surrounding the therapy sessions were lost. Conversations were less serious, and therapy sessions seemed to lose direction, which made therapy more supportive rather than a tool for change. The reflections and working through that were an essential ingredient in in-person sessions did not take place remotely, and the non-verbal communication was lost. The patients had difficulties in maintaining their concentration and the therapy focus, which made them tired and frustrated. Both the emotional relationship and the working alliance were negatively influenced. The emotional presence was experienced as weakened, but some of the patients could find expressing their feelings easier in the absence of bodily co-presence. According to the patients, in-person presence contributed to their security and trust, whereas they felt that the therapists seemed different when working remotely: more easygoing and familiar, but also more solution-focused, supportive and unprofessional, less understanding and less therapeutic. Despite their persisting, mainly negative longitudinal experiences, the patients also stressed that telepsychotherapy gave them an opportunity to take therapy with them into their everyday lives when they were in their own homes during the sessions. They appreciated the possibility to continue their treatment despite the pandemic. This finding is consistent with previous research (Christensen et al., 2021; Leuchtenberg et al., 2022). Even in the long term, remote therapy turned into something other than therapy had been in the conventional in-person setting, and once back in the therapy room, the patients felt the therapy started anew.

### Difficulties in telepsychotherapy

To a large extent, these findings regarding long-term experiences of the transition to telepsychotherapy resemble the results in a previous study on patients’ more immediate experiences of the transition during the COVID-19 pandemic in Sweden (Werbart et al., 2022). Like the present study, the previous study showed that respondents experienced a loss of therapeutic rituals, a decrease in productive therapeutic work, impaired contact, and less emotional presence. In both studies, some participants reported aspects of feeling freer and finding it easier to express certain material in telepsychotherapy, as well as thinking that remote therapy had the advantage of being more accessible and adaptable. One difference is that the previous study reported a typical theme of technology as hindering. This was not found in the present study, in which the participants had a slightly more positive view on telepsychotherapy. This difference might be explained by continuous longitudinal adjustment over time to telepsychotherapy, both by the patients and their therapists. With time, increasing experience, and occasionally with several transitions between the in-person and remote therapy setting, the patients and their therapists might have become more familiar and better adapted to the digital format. Furthermore, as the COVID-19 pandemic had been going on for at least 2 years when the present study was conducted, the patients and their therapists might have become more acquainted overall with, and skilled in, digital communication. Accordingly, a study of the therapists’ experiences of forced transitions to telepsychotherapy (Ahlström et al., 2022) showed that they initially struggled with technical and safety issues. The loss of the therapy room and of access to non-verbal nuances contributed to impaired contact with the patients and more superficial conversations. The therapists experienced that the very nature of psychodynamic psychotherapy was affected, even if telepsychotherapy could give some new opportunities. One year later many of the difficulties remained, but the therapists had developed better coping strategies and were back to the therapy focus. Likewise, according to a survey among 1,450 psychodynamic and psychoanalytic therapists (Aafjes-van Doorn et al., 2022), in the initial period of transitions most therapists regarded remote therapy as less effective than the traditional in-person setting; they felt more tired, less competent, and less in contact with their patients. This finding can be related to the patients in our study experiencing the therapist as different at a distance. A survey following up the therapists 8 months later (Aafjes-van Doorn et al., 2022) showed that the therapists regarded remote therapy as more similar to the customary setting, whereas the patients in our study still regarded remote therapy 2 years after the outbreak of COVID-19 pandemic as something different from in-person therapy.

### Positive aspects of telepsychotherapy

In the present study, the respondents expressed gratitude that psychotherapy could continue during the pandemic, thanks to the digital format. The lockdowns and restrictions during the COVID-19 pandemic resulted in increased isolation for many (Faustino et al., 2020; Hwang et al., 2020; Pai and Vella, 2021), which might explain the thankfulness that the therapeutic relationship could be preserved, although in another form. A new finding in the present study, absent in the study of Werbart and co-workers (2022), was the theme *New opportunities*, which includes the reflections that telepsychotherapy has the advantage of enabling contact with the therapist more frequently and despite geographical distance. However, telepsychotherapy was also described as somewhat more relaxed, less intense, and less effective. These mixed views might reflect an ambivalent attitude toward psychotherapy among patients in our study, with on the one hand a wish to maintain the relationship with the therapist, but on the other hand a wish to avoid the more challenging aspects of closeness and hard therapeutic work. Furthermore, these mixed results might reflect the therapists’ experiences with patients with different personality orientations and attachment styles. Some recent studies indicate that patients with personality orientation around issues of relatedness/closeness and patients with attachment anxiety experienced more distress in remote therapy during the COVID-19 pandemic than patients with personality orientation around issues of autonomy/performance and patients with attachment avoidance (Aafjes-Van Doorn et al., 2021a,b; Békés and Aafjes-van Doorn, 2022; Werbart et al., 2022). Thus, the therapists’ parallel work with different patients could contribute to the co-occurrence of their more negative and more positive views of remote work. Still another contribution to these inconsistent views might be the therapists’ own experiences of therapy, psychotherapy training and longstanding clinical work in conventional in-person settings.

### Telepsychotherapy as something else

Some of our findings concern changes in the therapeutic boundaries in connection with the transitions between the standard and remote therapy format. The experiences of the therapy becoming more relaxed, the therapists becoming more self-disclosing, and the therapy starting to blend with everyday life are all examples of boundary crossings. Lemma (2017) claims that the transition of therapy to the digital format can in itself be viewed as a boundary crossing, and therefore it is important that the boundaries are redefined in accordance with the new situation. Some of the rituals in the in-person psychotherapy format that patients find helpful were lost after the transition to the remote format, such as traveling to the therapist’s office and back again, which had allowed time to reflect and process. Wiener (2021) points out that the absence of these journeys could be regarded as deficiencies of the therapeutic frame. The respondents in the present study also recounted how they had to find a new safe spot at home where they could sit during therapy, and thus they had to create therapeutic frames and be responsible for them on their own. Descriptions of how therapists started to act differently, with less professionalism, after the transition to telepsychotherapy could be an indication of therapists being struck by beginners’ anxiety, previously described by Ehrlich (2021).

Viewed from the perspective of attachment theory, the transition to remote therapy could be described as a challenge to psychotherapy as a secure base aimed at facilitating exploration of mental and relational processes. Indications of this are the findings regarding the decrease in depth in therapy, increased difficulties in approaching emotions, and the therapeutic process becoming inhibited or stalled. According to Talia et al. (2019), the patient’s attachment to the therapist is shown in the degree of their openness and autonomy in relation to the therapist. A survey among 719 patients (Békés and Aafjes-van Doorn, 2022) led to the conclusion that patients’ attachment avoidance and their perception that the real relationship is of lower quality predict their more negative attitudes toward remote therapy. The findings in the present study indicate decreased openness and autonomy in the patients, which might show that more effort needs to be made to develop security and trust in remote format therapy. This conclusion could have important implications for psychotherapy training and supervision in an era when hybrid psychotherapy formats are beginning to be increasingly common.

In our study, the patients were generally dissatisfied with the transitions to the remote sessions, even if they also saw new opportunities in telepsychotherapy, and they experienced relief returning to the in-person setting. Their typical experience was that the remote format led to increased difficulties in understanding themselves and the other person. Important means of communication such as body language, eye contact, facial expressions and emotional atmosphere diminished or became more difficult to interpret. These results are in line with Knight’s (2020) observation that telepsychotherapy suffers from the loss of important sources of interpersonal communication, such as body language, which means that the persons involved lose important information about each other. According to Knight (2020), the unplanned shift to “part-body-on-the-screen relating” from what was once “whole-body relating” can lead to gaps in the relationship between patient and therapist and could contribute to the two parties relating on a more primitive, suspicious level, with more misinterpretations of each other. Respondents in the present study reported thoughts about how remote therapy increased the occurrence of overinterpreting and misunderstanding the therapist in the absence of body language. This could have a negative effect on the therapeutic alliance, as the experiences of not being understood and seen by the therapist to the same extent as before could decrease the emotional bond with the therapist and contribute to the experience of less efficient therapeutic work. The respondents reported that they found it harder to explain their suffering in remote therapy and that both the therapist and the patient ran into more difficulties in detecting increased patient suffering. Accordingly, a single case study of changes in clinical process due to transition to remote therapy (Negri and Christian, 2022) showed that both patient and therapist were working harder to remain connected and communicate that they were present, but with limited emotional engagement. Thematic analysis of open questions in a survey among 133 Norwegian patients (Stänicke et al., 2022) revealed the patients’ experience that the remote work brought an emotional distance to therapy, even if transitions to remote sessions were regarded as good enough emergency solutions, providing access to continuing therapy. In line with this, a Danish qualitative study using interviews and focus groups (Christensen et al., 2021) showed that both older patients with depression and their care providers regarded videoconferencing as a technological aid best suited for shorter follow-up meetings, and both groups stressed the need to establish in-person contact prior to remote sessions.

Thus, a relevant question is the concord or discord between the patients’ and therapists’ views of the benefits and drawbacks of remote sessions as compared to the in-person setting. In an online survey of patients and therapists in CBT after the first lockdown in Germany (Leuchtenberg et al., 2022), both groups regarded remote work as more flexible regarding the place and time of the sessions, but less helpful regarding the content of the therapeutic work, especially in cases of more complex problems and courses in therapy. The technical challenges of videoconferencing were experienced as more disturbing by the providers with negative expectations than by patients grateful for the possibility of continuing their treatments despite lockdown. Furthermore, patients experienced therapeutic alliance and empathy as comparable in videoconferencing and in face-to-face sessions, whereas therapists indicated advantages of in-person work. In a Danish qualitative study of patients in mental health services (Moeller et al., 2022), the seven participants experienced remote sessions as useful, and they could maintain good therapeutic relationships online when they had initially met their therapists in person. On the other hand, an Italian study of 23 patients and their five therapists in hybrid settings (Sperandeo et al., 2021) showed that the patients rated their therapists as significantly more empathetic and supportive in the remote sessions than in the in-person sessions, whereas the therapists experienced no such differences. In addition, the concordance between patient and therapist ratings was higher in the remote sessions than in the in-person sessions.

Many positive experiences of teletherapy are presented also in our study. However, both patients and therapists seem to show a more negative attitude to telepsychotherapy in in-depth interviews such as our study, even when reporting their long-term-term experiences (Dores et al., 2020; Ehrlich, 2021; Ellman and Vorus, 2021; Essig and Russell, 2021; Isaacs Russell, 2021; Ahlström et al., 2022; Reilly et al., 2022), than in surveys and rating scales (Békés and Aafjes-van Doorn, 2020; Mancinelli et al., 2021; Sperandeo et al., 2021; Aafjes-Van Doorn et al., 2021a,b; Farber and Ort, 2022). Such discrepancies between qualitative and quantitative studies have also been observed in outcome research (Desmet et al., 2021).

Furthermore, the difference between the generally negative long-term patient attitudes toward telepsychotherapy in our study and the positive experiences of therapists’ empathy and support in the Italian study (Sperandeo et al., 2021) may be due to differences in handling the COVID-19 pandemic. The Public Health Agency of Sweden (Folkhälsomyndigheten [The Public Health Agency of Sweden], 2020) recommended in March 2020 homework when possible, without such extensive lockdown as for example in Italy. Thus, the contrast between the remote psychotherapy setting and the more open social life was larger in Sweden, which could contribute to the more negative views. On the other hand, several interview studies from countries with more extensive lockdown had shown equal patient and therapist dissatisfaction with remote psychotherapy setting.

Some studies have shown that patients tended to be more satisfied with the transition to the remote setting than therapists, perhaps due to their gratitude for the possibility to continue treatment during the lockdown and to continue their treatment despite the pandemic (Christensen et al., 2021; Leuchtenberg et al., 2022), or to the therapists’ worries about preserving the integrity of treatment and about their ability to maintain their therapeutic stance (Thomas et al., 2021; Ahlström et al., 2022). The common features and differences between the patient and the therapist perspective on transitions to and from the remote setting are still underexplored and need further investigation. In our parallel study of therapists’ long-term experiences of telepsychotherapy following the COVID-19 pandemic (under review), the therapists still underlined the differences between the remote and in-person setting, and they stressed the need of acquiring new technical and relational skills. A learning from the present study might be that the patients need the therapists to adjust their interventions to the remote setting and to actively address the loss of the intermediate space and time between therapy sessions and the patient’s everyday life, as well as to make the altered emotional relationship an explicit therapeutic topic, and to contribute to distance regulation in the remote setting.

### Limitations and further directions

As the aim of this study was to increase understanding about how patients experienced the transition to telepsychotherapy during the COVID-19 pandemic, a qualitative approach with inductive thematic analysis was considered an adequate methodological choice. With this explorative aim, we found that semi-structured interviews with open questions, complemented by follow-up questions, was an appropriate form of data collection. We regard the sample size of 11 patients as a compromise between conducting an in-depth exploration of the participants’ experiences and striving to include participants with different therapeutic orientations and work conditions, while still allowing us to reach a saturation point when additional data fail to generate new understanding (Hennink et al., 2017; Braun and Clarke, 2022). Furthermore, this sample size is suitable for experiential thematic analysis and a study in a large project (Braun and Clarke, 2013, pp. 45, 49). The participants were in treatment with different theoretical orientations, varying time in therapy prior to the first transition and varying treatment duration, and using different communication aids. Such a heterogeneous sample can be seen as a limitation; however, our aim was to explore different facets of the patients’ long-term experiences of shifts between the in-person setting and remote sessions. It is a limitation that only one of the respondents from the previous interview study (Werbart et al., 2022) agreed to participate in the present study, as the original goal of investigating changes from immediate experiences to long-term experiences among patients who had transitioned to telepsychotherapy could not be completely fulfilled. Still another weak spot is that we could not include the patients’ therapists and explore similarities and differences of views within the therapeutic dyads. The present study was limited to the participants’ subjective perspectives and did not include quantitative measures of patient satisfaction, expectations, working alliances, or experienced outcomes. Furthermore, it might be a limitation that the interviews were conducted in digital format using Zoom, with the cameras turned off. As in telepsychotherapy, important interpersonal information from body language, facial expressions and eye contact became lost in the research interviews, which might have negatively affected the interview relationship.

Psychotherapists have had to adjust their interventions to telepsychotherapy, often in improvised form, since they were forced to switch to remote therapy. An area for further research is how therapists have modified their approach and interventions in order to overcome difficulties in the therapeutic relationship and intensity of treatment, as well as how patients experience these modifications. Some of the problems of telepsychotherapy that the present study pointed out might be possible to overcome, whereas others will not. This is something that research could find out. A further area of research is the increasingly common use of hybrid approaches, with one question being for which patients, under which circumstances and in which therapeutic modalities hybrid treatments can be justified, and when they are rather an expression of the patient’s or the therapist’s resistance and defenses.

## Conclusion

The present study of the patients’ experiences of switching between in-person and remote psychotherapy sessions contributes to several learnings for the therapists and researchers. Our results indicate that format alternations have an impact on which interventions can be implemented remotely or in hybrid treatments. Furthermore, there may be specific risks associated with the remote setting for patients with certain types of difficulties. For example, some patients did not dare to use remote therapy in the same way as in the conventional in-person setting due to their fears of not getting enough support and of increased self-harm and dissociation. On the other hand, for some patients the remote setting could facilitate the regulation of closeness and distance in the therapeutic relationship and the expression of their emotions. Another learning is that the therapists need to actively negotiate the transitions between in-person and remote sessions together with the patient. Exploring and working through patient experiences of format alternations might in itself become an important contribution to the therapeutic process. However, more knowledge is still needed to understand how in remote or hybrid settings the different therapeutic approaches have to be adapted to the patients’ problems and their individual needs for distance and closeness in relationships. A further topic for research is the role of the therapist in making the transition as helpful as possible.

## Data availability statement

The datasets presented in this article are not readily available because Interviews with participants cannot be shared due to confidentiality. Requests to access the datasets should be directed to camilla.vonbelow@psychology.su.se.

## Ethics statement

The studies involving human participants were reviewed and approved by Swedish Ethical Review Authority (registration numbers 2020-06819 and 2021-01188). The patients/participants provided their written informed consent to participate in this study.

## Author contributions

CB was responsible for this particular study and article within the larger project and designed the data collection and research questions. She supervised JB and TM in the process of the analysis, wrote early drafts of the article and developed these together with the other authors. JB and TM participated in designing data collection and were responsible for recruiting the participants, acquisition of all the data included, primary analysis and interpretation of the data for the work, early drafting, and critical revision in the later stages of the work. BP was co-researcher in the research project and participated in planning and designing the present study. He drafted some parts of the manuscript and participated in the text revisions leading to the final version to be submitted. AW was project leader and principal investigator in the research project on transitions to telepsychotherapy. He participated in planning and designing the present study, continuously scrutinized the progress of the study, data analysis, interpretation of results, and early drafting, and substantially contributed to the final version to be submitted. All authors have given final approval of the version to be published and agreed to be accountable for all aspects of the work.

## Funding

The authors disclosed receipt of the following financial support for the research, authorship, and or publication of this article: The present study is a part of the research project *Transitions to telepsychotherapy and ways back to the office, personality orientation and attachment style: Long-term effects of COVID-19 pandemic on provision of psychotherapy*, awarded by the Board of Human Science, Stockholm University, registration number SU FV-5.1.2-3314 -20, by the Fund for Psychoanalytic Research of the American Psychoanalytic Association, dated May 26, 2022, and by the International Psychoanalytical Association Research Grant dated October 12, 2022.

## Conflict of interest

The authors declare that the research was conducted in the absence of any commercial or financial relationships that could be construed as a potential conflict of interest.

## Publisher’s note

All claims expressed in this article are solely those of the authors and do not necessarily represent those of their affiliated organizations, or those of the publisher, the editors and the reviewers. Any product that may be evaluated in this article, or claim that may be made by its manufacturer, is not guaranteed or endorsed by the publisher.
